# Salt Losing Obstructive Uropathy with Paradoxically Low Urinary Sodium Concentration: Salt Entrapment in an Obstructed Ectopic Ureterocele

**DOI:** 10.5402/2011/453271

**Published:** 2011-03-29

**Authors:** Steven M. Zangan, David K. Yousefzadeh

**Affiliations:** Department of Radiology, The University of Chicago Medical Center, 5841 S. Maryland Avenue, MC 2026, Chicago, IL 60637, USA

## Abstract

A 6-month-old hyponatremic female with failure to thrive had low urinary sodium concentration. Renal sonography revealed a duplex left collecting system with obstruction of the upper moiety as a blind-ended ectopic ureterocele extending to the bladder base. The echogenicity of the urine within the upper pole system was greater than the bladder contents. We believed that low urinary sodium concentration represented a false negative test and the salt loss by the obstructed left kidney was entrapped in the upper pole collecting system. Prior to ureterocele repair, intraoperative bladder and ureterocele aspirates revealed discordant sodium concentration supporting the sonographic conclusion.

## 1. Introduction

Though the most common presentation of obstructive uropathy is urosepsis [1], this disorder may be associated with hyponatremia and loss of salt in the urine. The present case posed a diagnostic challenge because despite the presence of an obstructive salt losing uropathy, the urinary salt concentration was paradoxically low. This previously unreported paradox was due to the entrapment of a high concentration of salt within the obstructed upper moiety of a duplex system, the contents of which were not mixing with urine in the bladder. This resulted in a falsely low sodium concentration on urinalysis.

## 2. Case Report

### 2.1. Admission 1

An afebrile 6-month-old female was transferred to our institution with lethargy, vomiting, and diarrhea. Laboratory evaluation was remarkable for metabolic acidosis, hyponatremia (133 mEq/L), and hypokalemia (3.4 mEq/L). Since the patient promptly improved and laboratory values, including urinary sodium concentration, corrected with intravenous hydration, the patient was discharged with the diagnosis of dehydration without a proper work-up for obstructive uropathy. 

### 2.2. Admission 2

**Figure 1:**
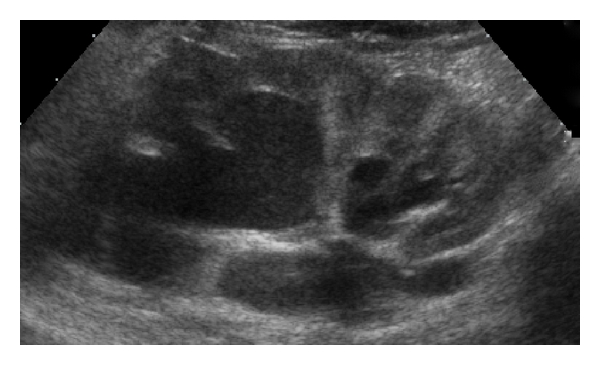
Left renal sonogram demonstrates a duplicated collecting system. Note the dilation of the upper pole moiety.

**Figure 2:**
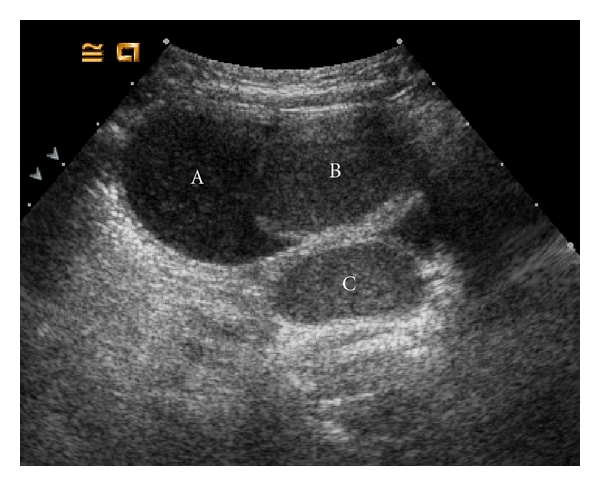
Bladder sonogram. The echogenicity of the urine within the bladder (A) differs from that within the ureterocele (B) and obstructed upper pole ureter (C).

**Figure 3:**
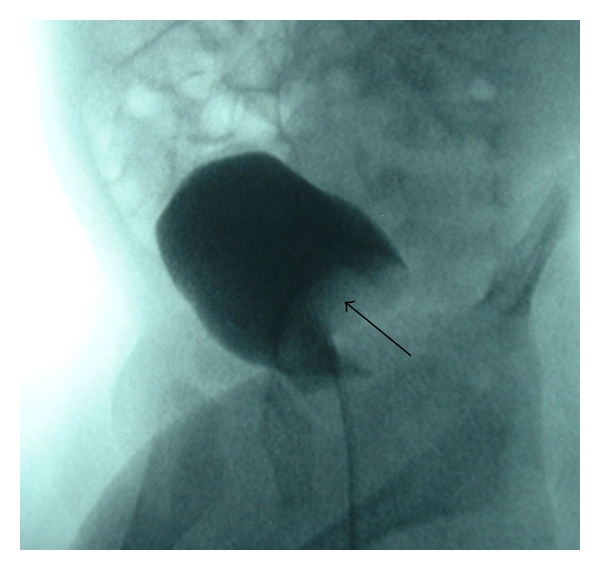
Characteristic large intravesical filling defect (arrow) on voiding cystourethrogram consistent with ureterocele.

Three weeks later, the patient was readmitted with failure to thrive. Admission laboratory work revealed metabolic acidosis, hyponatremia (123 mEq/L), hyperkalemia (6.0 mEq/L), and decreased serum osmolality (270 mOsm/kg). The urine sodium and osmolality were also decreased, 10 mEq/L and 101 mOsm/kg, respectively. The patient was afebrile and urine cultures were negative. Plasma renin was >1800 mcU/mL (normal 6–64 mcU/mL) and aldosterone was 1621 ng/dL (normal 7–99 ng/dL), both markedly elevated. There was no history of significant vomiting or diarrhea and the cause of the electrolyte abnormalities could not be explained. Renal sonography demonstrated a duplex left collecting system (Figure 1) and dilation of the upper pole moiety terminating as a large ectopic ureterocele at the bladder base. The echogenicity of the trapped urine was homogeneously increased suggesting a higher concentration of debris or minerals (Figure 2). Voiding cystourethrography confirmed the presence of a ureterocele and was otherwise normal (Figure 3). The sonographic findings and discordance between urine and serum sodium concentration suggested that the enclosed upper pole collecting system had entrapped a higher sodium concentration, not reflected in the urinalysis. 

The patient was taken to surgery for cystoscopic transurethral incision of the obstructing ureterocele. Prior to repair, aspirates of the bladder and ureterocele revealed discordant sodium concentration, 22 mEq/L and 118 mEq/L, respectively. The patient had an uncomplicated postoperative course and improved with supportive care.

## 3. Discussion

Ectopic ureterocele is associated with ureteral duplication and obstructive dilation of the ureter and its calices. Though the most common presentation is urinary tract infection, some children have a more insidious course with intermittent abdominal pain or failure to thrive. Rarely, the infants have salt losing obstructive uropathies. Salt losing nephropathy associated with obstructive uropathy has been called pseudohypoaldosteronism and is thought to reflect tubular unresponsiveness to aldosterone [2]. Though the combination of renal tubule immaturity, obstructive uropathy, and urinary tract infection are predisposing factors, pseudohypoaldosteronism has been described with pyelonephritis independent of obstruction [3] and with obstruction independent of infection [4]. Because of these associations, urine culture and renal sonography have been advocated in any infant with an electrolyte disturbance [5].

Ordinarily the salt concentration in a urine sample correlates with the degree of renal salt excretion. However, our case demonstrates that anomalies resulting in entrapment or diversion of the urine with high salt concentration away from the bladder create exceptions to this principle. The paradox exemplified here need not be limited to cases associated with an obstructed ectopic ureterocele terminating at the bladder base. If the ectopic ureter of an obstructed moiety enters anywhere else than the bladder, such as the seminal vesicles, vagina, or urethra, similar clinical challenges may be encountered. Though the observation of echogenic fluid within a ureterocele has been made before [6], it is usually attributed to nonspecific debris or pus related to urinary tract infection. In our case, which was not associated with urosepsis, sonography helped explain the unusual laboratory results. 

Normal or decreased urinary sodium concentration and hyponatremia do not exclude salt losing obstructive uropathies in patients with hyponatremia. The excess salt can be entrapped in an obstructed system resulting in a false-negative urinalysis and diversion of attention away from this surgically correctible group of disorders.

## References

[1] Coplen DE, Duckett JW (1995). The modern approach to ureteroceles. *Journal of Urology*.

[2] Levin TL, Abramson SJ, Burbige KA, Connor JP, Ruzal-Shapiro C, Berdon WE (1991). Salt losing nephropathy simulating congenital adrenal hyperplasia in infants with obstructive uropathy and/or vesicoureteral reflux—value of ultrasonography in diagnosis. *Pediatric Radiology*.

[3] Gerigk M, Glanzmann R, Rascher W, Gnehm HE (1995). Hyponatraemia and hyperkalaemia in acute pyelonephritis without urinary tract anomalies. *European Journal of Pediatrics*.

[4] Terzi F, Assael BM, Claris-Appiani A (1990). Increased sodium requirement following early postnatal surgical correction of congenital uropathies in infants. *Pediatric Nephrology*.

[5] Bulchmann G, Schuster T, Heger A, Kuhnle U, Joppich I, Schmidt H (2001). Transient pseudohypoaldosteronism secondary to posterior urethral valves—a case report and review of the literature. *European Journal of Pediatric Surgery*.

[6] Fernbach SK, Feinstein KA, Spencer K, Lindstrom CA (1997). Ureteral duplication and its complications. *Radiographics*.

